# Comment on Lai et al. Liver Transplantation for the Cure of Neuroendocrine Liver Metastasis: A Systematic Review with Particular Attention to the Risk Factors of Death and Recurrence. *Biomedicines* 2024, *12*, 2419

**DOI:** 10.3390/biomedicines14092109

**Published:** 2026-09-18

**Authors:** Zeynep Dal, Mert Erkan

**Affiliations:** 1 Department of General Surgery, Heidelberg University Hospital, 69120 Heidelberg, Germany; zeynep.dal@med.uni-heidelberg.de; 2 Department of General Surgery, Faculty of Medicine, Acibadem Mehmet Ali Aydinlar University Hospital, Istanbul 34642, Turkey

**Keywords:** gastrointestinal neuroendocrine tumors, NET-associated liver metastases, living donor liver transplantation

## Abstract

Gastrointestinal neuroendocrine tumors (GI-NETs) are being diagnosed with increasing frequency and often manifest with liver metastases, presenting considerable therapeutic challenges. This commentary addresses the systematic review by Lai et al., which evaluated liver transplantation (LT) for NET-associated liver metastases (NEN-LM). Although Lai et al. identified LT as a promising intervention for unresectable or liver-confined NETs, we believe that certain aspects warrant further discussion. To begin with, approximately 16% of reported cases had an unknown primary tumor. This not only complicates patient selection and potentially introduces confounding outcomes but also contradicts the notion of “liver-limited disease”. Furthermore, elevated proliferative activity, as indicated by a Ki-67 index of >2%, has been correlated with reduced survival rates. Therefore, LT should ideally be offered to patients with GI-NETs with a Ki-67 index of <2%. This subgroup of patients may also benefit from alternative therapy options, such as cytoreductive surgery/tumor debulking combined with peptide receptor radionuclide therapy (PRRT), which has demonstrated favorable outcomes and deserves greater attention. Finally, advocating LT for patients with NEN-LM based on retrospective data might facilitate its overuse, especially in countries/regions where living donor LT is employed. We believe that this important yet missing point merits an in-depth examination of its advantages and disadvantages.

## 1. Introduction

Neuroendocrine tumors (NETs) of the gastrointestinal tract are a heterogeneous group of neoplasms that arise from neuroendocrine cells distributed throughout the digestive tract [1,2]. These tumors exhibit significant variation in their origin, level of differentiation, rate of growth, hormone secretion, and clinical manifestations, resulting in a spectrum of disease rather than a single, uniform condition. Some can be functional and secrete bioactive substances that lead to specific syndromes, while some are nonfunctional and come to attention only through nonspecific symptoms or incidental detection. Many are well differentiated and may follow a prolonged clinical course, yet they retain malignant potential and can present with regional or distant spread [1,2]. Among these patients who present with nodal or distant metastases, hepatic involvement is the most frequent manifestation [3].

Broad heterogeneity has long been recognized as a central feature of gastrointestinal neuroendocrine tumors (GI-NETs) [4]. Management of both primary tumors and NET-associated liver metastases (NEN-LM) remains an area of ongoing multidisciplinary debate. Current strategies typically begin with somatostatin analogs for well-differentiated somatostatin receptor (SSTR)—positive tumors and escalate to peptide receptor radionuclide therapy (PRRT) as clinically indicated. When significant cytoreduction is achievable, commonly defined as ≥70%, liver-directed surgery or ablation using parenchyma-sparing techniques is considered [3,5]. For patients with unresectable, liver-dominant disease, radioembolization can provide palliation and disease control, while liver transplantation has been explored as an alternative, with candidacy often guided by the Milan criteria for NEN-LM [6]. Overall, treatment selection remains dynamic and evolving, requiring careful consideration of both the established and emerging therapeutic options.

## 2. Key Findings Analysis

The systematic review by Lai et al. synthesizes literature published since 2000 on liver transplantation (LT) as a treatment for patients with neuroendocrine neoplasm liver metastases (NEN-LM), with particular focus on risk factors, mortality, and recurrence [7]. The review encompasses 27 retrospective studies from 13 countries, reporting 5-year overall survival (OS) rates after LT ranging from 52% to 74%, and 5-year tumor-free survival rates between 39% and 62%, thus suggesting that LT can be a potentially effective therapeutic option in carefully selected patients.

The authors highlighted that the outcomes of LT for NEN-LM have improved notably, particularly since the year 2000. They classified the prognostic factors for LT into distinct categories. According to the Milan criteria, patients with a Ki-67 index of ≤2% (G1) are deemed suitable candidates for LT. In alignment with this, the authors indicated that elevated Ki-67 indices and higher tumor grades are linked to increased risks of mortality and recurrence. Concerning metastatic liver involvement, extensive hepatic involvement exceeding 50% has been associated with less favorable outcomes, and hepatomegaly has also been recognized as a significant risk factor.

Regarding primary tumor characteristics, the need for multivisceral transplantation, simultaneous resection of the primary tumor during LT, and the need for major extrahepatic resection were associated with decreased survival rates. Factors related to transplantation, such as advanced recipient age, prolonged cold ischemia time, and elevated Model of End-Stage Liver Disease (MELD) scores, were similarly linked to poorer outcomes. Based on limited data, the authors suggest that LT may offer improved survival and reduced recurrence risk compared with resection alone. Compared to other tumor types, patients with NET exhibit either lower recurrence rates or comparable 5-year overall survival (OS) following LT.

Taken together, the authors propose that the established selection criteria for LT candidates, such as the Ki-67 threshold and age limits, may warrant reconsideration and potential expansion.

## 3. Critical Evaluation

While this comprehensive systematic review provides valuable insights into the application of liver transplantation (LT) in patients with neuroendocrine neoplasm liver metastases (NEN-LM), several critical aspects merit further discussion.

Liver transplantation encompasses both deceased donor and living donor liver transplantation (DDLT and LDLT) procedures. However, this review did not specifically analyze or differentiate LDLT, likely due to inconsistent reporting within predominantly deceased donor–based retrospective studies, which limits insights into LDLT. This omission is particularly notable given LDLT’s significant clinical relevance in several countries where it constitutes a substantial proportion of liver transplants. Therefore, when considering transplantation as a treatment option, it is crucial to recognize the distinct clinical considerations associated with both LDLT and deceased donor transplantation. While LDLT poses risks to the donor and is generally reserved for cases where the benefit is well-established and maximized, deceased donor transplantation faces challenges such as organ availability and long waiting lists. These factors must be carefully considered to optimize patient outcomes and resource allocation.

Liver transplantation for NEN-LM has been studied primarily in deceased donor series, and data for direct comparison with LDLT outcomes remain limited. Reported 5-year overall survival rates have ranged from 36% in early European cohorts [8] to 57–65% in larger registry analyses [9,10,11]. In contrast, research on living donor liver transplantation (LDLT) for NEN-LM remains limited to isolated case reports and small national series, with most data originating from Turkey and Japan. Two emergency LDLT cases reported survival durations of 34 months and disease-free status at 45 months post-transplant [12,13], and a pancreatic insulinoma case achieved 13-year survival after simultaneous distal pancreatectomy and LDLT [14]. Furthermore, a small Japanese cohort study involving 16 patients demonstrated a 5-year OS rate of 50% [15]. However, the small sample sizes and limited reporting preclude definitive conclusions or meaningful comparison with deceased donor outcomes.

A further notable limitation is that approximately 16% of the patients (out of 381 across 27 studies) had primary tumors that were unknown or undetected. Remarkably, even after major surgical intervention, the primary tumor site remains unknown in approximately one out of every six cases. This uncertainty may influence patient selection, outcomes, and risk stratification and challenges the term “liver limited,” although the review does not present specific analyses or outcome data for this subgroup.

Various therapeutic strategies beyond LT have emerged as relevant considerations in this context. In cases in which curative resection is not feasible, cytoreductive surgery may alleviate symptoms and improve patient outcomes. Achieving a tumor reduction of ≥80–90% has been associated with both symptomatic relief and increased survival rates, whereas even less extensive debulking (~70%) can still provide clinical benefits for selected patients [16]. Resection of primary pancreatic NETs (pPanNEN) and metastatic disease have also been linked to improved survival outcomes. A meta-analysis demonstrated a reduced risk of mortality in patients who underwent pPanNEN resection compared to those who did not, with a weighted 5-year overall survival (OS) difference of 32% [17]. It is notable to acknowledge, however, that prior cytoreductive surgery may increase the technical complexity of subsequent liver transplantation, as evidenced by higher intraoperative transfusion requirements and greater surgical demands reported in patients with a history of prior abdominal surgery [18]. While this does not represent an absolute contraindication, it underscores the importance of early multidisciplinary evaluation, so that both treatment pathways can be presented to the patient and decision-making guided by which approach is expected to provide the greatest overall benefit.

For SSTR-positive well-differentiated NETs with liver metastases, cytoreductive surgery is generally considered when meaningful tumor reduction is achievable. Nevertheless, even with a debulking of ≥70%, the median liver progression-free survival remains limited (~11 months), and the recurrence rates are notably high (~80% within 3 years). Peptide receptor radionuclide therapy (PRRT) has emerged as an effective treatment strategy that utilizes radiolabeled somatostatin analogs, most commonly lutetium-177 (^177^Lu)-DOTATATE, to deliver targeted radiation to tumor cells expressing somatostatin receptors, thereby achieving cytotoxic effects while minimizing damage to the surrounding tissues. This treatment has been reported for patients with advanced NETs, particularly following the landmark NETTER-1 trial, which demonstrated its efficacy in progressive midgut NETs [19,20]. In the neoadjuvant context, PRRT has demonstrated efficacy in reducing tumor size sufficiently to permit curative resection in cases previously deemed inoperable, with promising results for both primary tumors and metastatic disease [21,22,23]. A cohort study involving 94 patients with G1-G2 pancreatic NETs and unresectable liver metastases demonstrated that resection of the primary tumor prior to PRRT in patients with unresectable liver metastases enhanced progression-free survival (70 vs. 30 months) and overall survival (112 vs. 65 months) compared with PRRT alone, highlighting the advantage of combining debulking surgery with PRRT [24].

Beyond its neoadjuvant role, PRRT may offer particular potential in the adjuvant context after cytoreductive surgery. Post-debulking, the diminished tumor burden results in a reduced somatostatin receptor distribution volume, potentially enabling PRRT to deliver a more concentrated therapeutic effect to the residual disease, thereby enhancing its ablative capacity. This rationale parallels the use of radioactive iodine (RAI) following thyroidectomy for differentiated thyroid cancer, where post-surgical RAI targets remnant thyroid tissue, although the clinical benefit of this approach remains debated, with recent evidence questioning its necessity in low-risk patients [25,26,27]. Similarly, evidence supporting the use of adjuvant PRRT in patients with NEN-LM remains limited. Available data support its application primarily in metastatic and post-debulking settings [28,29], while adjuvant somatostatin analogs (SSAs) have shown potential benefits in select high-risk G2 pancreatic NETs [30,31,32] and type 1 gastric NETs [33], although a large mixed cohort found no benefit with unselected adjuvant therapy [29,34]. Nonetheless, PRRT presents a compelling rationale for its adjuvant application following cytoreduction, as residual microscopic disease with intact somatostatin receptor expression may be particularly susceptible to targeted radionuclide ablation. Therefore, while routine adjuvant PRRT or SSA use remains experimental outside high-risk settings or clinical trials, prospective investigations are crucial to determine whether post-cytoreduction radionuclide therapy provides significant benefits for patients with NEN-LM.

The risks and ethical considerations associated with living donor liver transplantation (LDLT) are well recognized. However, in many countries, LDLT is pursued not by choice but out of necessity, especially in situations where deceased donor liver transplantation (DDLT) is not the primary practice. As a result, LDLT often stands as the only viable transplantation option for eligible patients. Nonetheless, advocating for liver transplantation in patients with NEN-LM based on retrospective data may lead to its overuse in settings where LDLT accounts for a significant portion of transplant activity. On the other hand, deceased donor liver transplantation carries its own ethical constraints. Deceased donor availability varies substantially across regions. Spain reported 48 deceased donors per million population in 2022, whereas countries such as Turkey, where LDLT accounts for over 90% of all liver transplants performed, face considerably more limited deceased donor resources [35]. Where deceased donor availability is severely constrained and organ allocation is governed by national or regional authorities that generally do not prioritize patients with metastatic disease, an additional layer of complexity specifically affects the patient cohort discussed in this commentary, one that warrants careful consideration in future clinical guidelines and prospective research frameworks.

It is crucial to acknowledge that the subset of patients who fulfill the Milan criteria for LT is exceptionally limited. As previously mentioned, approximately 16% of reported cases involve primary tumors of unknown origin, and the strict criteria—including Ki-67 ≤2%, <50% liver involvement, prior curative resection of the primary tumor, and disease stability for at least six months—substantially limit the eligibility. Promoting LT as a promising therapeutic option raises substantial concerns, particularly in regions where living donor liver transplantation (LDLT) constitutes a significant proportion of the transplant activity. Unlike deceased donor transplantation, LDLT exposes healthy donors to considerable surgical risks, which can only be ethically justified when the clinical benefit is firmly established. Encouraging LT for NEN-LM and potentially expanding indications without robust evidence may inadvertently facilitate overuse and place donors at unnecessary risk. This critical consideration warrants a thorough discussion alongside any proposed benefits of transplantation.

## 4. Conclusions

Patient selection remains critical when considering LT for NEN-LM, as only a small subset of patients meets the stringent Milan criteria. The limited and predominantly retrospective evidence, the risk to healthy donors in living donor LT, and the notable proportion of unknown primary tumors all underscore the need for caution in generalizing these reported outcomes. In regions where living donor liver transplantation (LDLT) is prevalent, advancing liver transplantation (LT) without prior prospective validation may subject healthy donors to unnecessary surgical risks. Meanwhile, it is essential to further investigate alternative strategies, such as cytoreductive surgery, in conjunction with PRRT.

## Data Availability

No new data were created or analyzed in this study. Data sharing is not applicable to this article.
